# Supplementary material for: Is Boccia XR an enjoyable and effective rehabilitation exercise for older adults?
Source: PLoS One. 2025 Apr 4;20(4):e0320369. doi: 10.1371/journal.pone.0320369 (PMC11970655; doi:10.1371/journal.pone.0320369)
Supplement: Attachment — Submitted filename: Response to Reviewers.docx [file pone.0320369.s006.docx]

**Hasan Sözen**

**Academic Editor**

**PLOS ONE**

Submission title: Is Boccia XR an enjoyable and effective rehabilitation exercise for older adults?

Manuscript ID: PONE-D-24-42948

Dear Hasan Sözen, Academic Editor,

Thank you for providing us with the reviewers’ comments regarding our manuscript titled “Is Boccia XR an enjoyable and effective rehabilitation exercise for older adults?”. We appreciate the valuable feedback and have carefully considered all the points raised. Below, we provide detailed responses to each comment and outline the revisions made in the manuscript.

**Corrections to Data Processing**

Before addressing the specific comments from the reviewers, we would like to note two important corrections made to our dataset:

**1. Correction to the POMS Data:**

We identified that one participant's data was missing in the POMS analysis, resulting in an analysis based on 17 participants instead of the intended 18. We have now corrected this to ensure that all 18 participants are included.

**2. Correction to the EMG Analysis for the Rectus Femoris in the XR Condition:**

An error was found in the data processing window for the Rectus Femoris EMG measurement in the XR condition. This has been corrected to reflect the intended analysis window.

**Importantly, these corrections did not alter the statistical significance of the results or change our main conclusions. The discussion section remains consistent with the original interpretation, and only minor numerical adjustments have been made in the Results section.**

**Note on Page and Line Numbers**

For clarity, all page and line numbers provided in our responses refer to the final revised version of the manuscript without tracking changes. This ensures accurate reference points, as line numbers may differ in the tracked changes version.

**Summary of Revisions**

We have addressed all the comments raised by the reviewers, including clarification of the methodology, additional statistical analyses, and a more detailed discussion of the study’s limitations and future directions.

Below, we provide point-by-point responses to each comment and specify the corresponding revisions.

Sincerely,

Masataka Kataoka

Osaka Metropolitan University, Graduate School of Rehabilitation Science

**Reviewer #1:**

**General Feedback:**

“The study presents findings from original research and demonstrates its significance... The exploration of this population group addresses important gaps in the literature and contributes meaningfully to advancing knowledge in the field.”

**Response:**

Thank you for your positive feedback. We appreciate your recognition of our efforts to address gaps in the literature and contribute to the field.

**Comment 1:**

“The introduction could be further enriched and expanded to provide a more comprehensive foundation for the study... A more thorough introductory reflection on these challenges, addressing both global and context-specific perspectives, would add significant depth and contextual relevance to the study.”

**Response:**

Thank you for your suggestion. We have expanded the introduction to address global and context-specific challenges faced by older adults and clarified key concepts.

**Revision:**

The following sentences have been added to the introduction on page 4, lines 42–48:

“This decline in physical activity is a major concern worldwide, as it contributes to reduced mobility, increased risk of falls, and overall decline in quality of life among older populations [6]. The World Health Organization (WHO) has emphasized the importance of promoting accessible and engaging physical activities for aging populations to prevent frailty and disability [7]. Additionally, psychological factors, such as social isolation and lack of enjoyment in traditional exercise routines, further hinder adherence to physical activity programs among older adults [6,8]”

On page 6, line 78–83:

“By integrating extended Reality (XR) technology, Boccia XR has the potential to enhance engagement and motivation in older adults, similar to the positive effects observed with Wii- and VR-based exercises [29–37]. Digital interventions are increasingly recognized as effective tools for promoting physical activity in aging populations, particularly among individuals who face barriers to conventional exercise [38].”

**Comment 2:**

“The sample size (N=18) and the participant selection process impose limitations... A discussion of the implications of the small sample size and its potential limitations would strengthen the methodological rigor.”

**Response:**

We acknowledge the limitations of the small sample size and have expanded the discussion in the Methodology and Discussion sections. The Participants section now mentions the use of GPower, while the Statistical Analysis section provides the rationale for the calculation.

**Revision:**

On page 8, lines 124–127, we corrected and added:

“A sample size calculation using G*Power software (Heinrich Heine University, Düsseldorf, Germany) determined that a minimum of 12 participants per condition was required. Further details on the power analysis are provided in the Statistical Analysis section.”

On page 20–21, lines 328–353, we added:

“To determine the appropriate sample size, an a priori power analysis was conducted using G*Power software. Previous studies on exergame interventions for older adults have reported moderate effect sizes (Cohen’s d = 0.54–0.65) in improving balance, physical activity, and psychological well-being [49-51]. However, some studies have raised concerns about usability and acceptance, suggesting that complex interfaces and unfamiliar technology may reduce motivation [52]. In contrast, Boccia XR is designed for intuitive use by older adults, eliminating the need for complex controllers or virtual environments. Its interactive and social nature is expected to enhance motivation and adherence in physical activity interventions. Given these advantages, we hypothesized that Boccia XR would have a greater impact than conventional exergames, potentially leading to a larger effect size. As no direct variance estimates were available from prior research, we adopted Cohen’s general guideline for behavioral intervention studies [53]. Based on this guideline, the sample size was calculated for a two-way repeated-measures ANOVA with an alpha level of 0.05, a power (1-β) of 0.80, and an effect size (f) of 0.40, which corresponds to a partial eta squared (ηp²) of approximately 0.14, classified as a large effect. The power analysis was based on expected differences in VA scores from POMS2, as mood enhancement was a primary outcome of interest. Given prior research on mood enhancement through physical activity, we anticipated a moderate to large effect size in VA scores, justifying the use of an effect size (f) of 0.40 for the analysis. The analysis indicated that a minimum of 12 participants per condition was required to achieve sufficient statistical power. Additionally, for comparisons conducted using the Friedman test, effect sizes were calculated using Wilcoxon’s r, following the formula:

r = Z / √N

The interpretation of Wilcoxon’s r followed Cohen’s criteria (0.1= small, 0.3= medium, 0.5= large), ensuring consistency in reporting effect sizes across different statistical methods.”

On page 29, lines 501–509, we included:

“The small sample size (n=18) limits the statistical power and generalizability of the results. Additionally, the study included only healthy older adults, which may limit the external validity of the findings, particularly for populations with different health conditions or functional limitations. Furthermore, none of the participants had prior experience playing Boccia, which may have minimized variability in baseline skill levels but could also have influenced familiarity with the game. Future studies with larger and more diverse populations, including individuals with varying health conditions, are needed to validate these findings and explore the long-term effects of Boccia XR on exercise adherence.”

**Comment 3:**

“More detailed explanations of the participant selection criteria, measures and instruments used, and steps to minimize bias would enhance transparency and rigor.”

**Response:**

Thank you for your suggestion. We have clarified the participant selection criteria and provided a more detailed description of the randomization process for transparency. The revised Intervention section includes an explanation of block randomization. Additionally, we have refined the description of POMS2 in the 'Mood State Measure' section to highlight its suitability for older adults.

**Revision:**

On page 8, lines 119–122, we revised the text as follows:

“Participants met the following inclusion criteria: (1) aged 65 years or older, (2) able to walk independently without assistive devices, and (3) able to understand and follow instructions. Recruitment was conducted without incentives to minimize bias.”

On page 9, lines 136–138, we added:

“Block randomization was used to counterbalance task order. Participants were divided into three blocks of six, each beginning with a different task. Subsequent tasks were balanced within blocks to ensure even distribution.”

On page 14, line 227–229, we revised as follows:

“POMS can measure mood changes over short periods [18, 43, 44] and is considered an appropriate tool for evaluating mood in older adults [41].”

**Comment 4:**

“Improving the clarity, fluency, and academic rigor of the language would enhance the overall readability value of the article.”

**Response:**

Thank you for your feedback. We have revised the manuscript to improve clarity, fluency, and academic rigor by removing redundant expressions and refining the language for better readability and scientific accuracy.

**Comment 5:**

“Expanding on the randomization process for participant selection... would also improve the overall quality of the research.”

**Response:**

Thank you for your comment. We acknowledge the importance of detailing the randomization process. As addressed in our response to Comment 3, we have added a more comprehensive explanation in the Methodology section.

**Revision:**

On page 9, lines 139–141, we added:

“Block randomization was used to counterbalance task order. Participants were divided into three blocks of six, each beginning with a different task. Subsequent tasks were balanced within blocks to ensure even distribution.”

**Final Note:**

We appreciate your thoughtful feedback. We believe our revisions comprehensively address your comments, but we remain available for further clarification if needed.

**Reviewer #2:**

**Comment 1: Novelty**

“This study compares Boccia XR with traditional Boccia and treadmill walking, yielding intriguing results. However, have comparisons with other exercise programs for older adults (e.g., Wii Fit, VR-based training) been considered? Virtual reality exercise programs, in general, may be enjoyable and health-promoting. Demonstrating Boccia XR's advantages over these programs would further enhance the value of this research. Even if this is addressed in future studies, please refer to past research on other virtual reality exercise programs and clarify the novelty of this study.”

**Response:**

Thank you for your insightful comment. We have clarified in the Introduction how Boccia XR differs from conventional VR-based interventions and further elaborated on this in the Discussion. Specifically, Boccia XR retains real-world physical interactions, making it more accessible for older adults than fully immersive VR systems. Moreover, since it does not require a head-mounted display, it eliminates the risk of motion sickness. Additionally, we suggest in the Discussion that future studies compare Boccia XR with immersive VR exercise programs to better assess its benefits.

**Revision:**

On page 6, line 83–90:

“Many VR-based exercise programs, such as Wii Fit and immersive VR training, require head-mounted displays or motion controllers, which may limit accessibility for older adults. Additionally, previous studies have reported that immersive VR experiences can induce motion sickness, particularly in prolonged use [39, 40]. In contrast, Boccia XR eliminates concerns about motion sickness by not requiring a head-mounted display while preserving real-world physical interactions by allowing participants to throw actual balls, ensuring ease of use while incorporating digital engagement.”

On page 28, line 461–470:

“Additionally, many VR-based systems, including Wii Fit and immersive VR training, require head-mounted displays or motion controllers, which may limit accessibility for older adults unfamiliar with such technologies [54–57]. In contrast, Boccia XR retains real-world physical interactions by allowing participants to throw actual balls, ensuring ease of use while still incorporating digital engagement. While this study did not directly compare Boccia XR with other VR-based exercise programs, future research should consider investigating its effectiveness relative to fully immersive virtual exercise interventions. Such comparisons could provide deeper insights into the unique benefits of Boccia XR in enhancing motivation and physical activity adherence among older adults.”

**Comment 2: Sample and Population**

“Are the findings of this study applicable to older adults aged 65 and above who are capable of walking? Could there be a bias in recruiting participants already interested in Boccia or exercise? Were participants with prior experience in treadmill walking or Boccia included? Please explain the Population targeted in this study and how the sample was recruited. Furthermore, please elaborate on the rationale for setting a relatively large effect size of 0.40.”

**Response:**

Thank you for your comment. This study specifically targeted older adults aged 65 and above who were capable of walking, and all participants met this criterion. Given that all participants were community-dwelling older adults recruited through a general call for volunteers, our findings can be reasonably generalized to similarly active older adults.

Regarding participant recruitment, we have clarified the process and characteristics in the Methods section. While we did not set exclusion criteria based on prior treadmill walking or Boccia experience, no participants reported previous experience in playing Boccia during recruitment screening.

Additionally, we acknowledge that the self-selection process may have led to the inclusion of individuals more interested in physical activity, potentially introducing a participation bias. We have now explicitly discussed this limitation in the revised Discussion section.

Finally, we have elaborated on the rationale for setting a relatively large effect size of 0.40 in the revised manuscript.

**Revision:**

On page 8, lines 121–124, we added as follows:

“Recruitment was conducted without incentives to minimize bias. While participants with prior experience in treadmill walking or Boccia were not excluded, none had previously played Boccia.”

On page 30–31, lines 504–512, we included:

“The small sample size (n=18) limits the statistical power and generalizability of the results. Additionally, the study included only healthy older adults, which may limit the external validity of the findings, particularly for populations with different health conditions or functional limitations. Furthermore, none of the participants had prior experience playing Boccia, which may have minimized variability in baseline skill levels but could also have influenced familiarity with the game. Future studies with larger and more diverse populations, including individuals with varying health conditions, are needed to validate these findings and explore the long-term effects of Boccia XR on exercise adherence.”

**Comment 3: Boccia XR**

“The enjoyment of Boccia XR may depend on its user interface. What specific features or innovations are incorporated into Boccia XR? Does it include sound or vibration feedback, as in other video games? Is it designed to prioritize entertainment, making it simpler than traditional Boccia? To ensure the reproducibility of the research, please provide more detailed information about Boccia XR.”

**Response:**

Thank you for your comment. We have expanded the Methods section to provide a clearer description of Boccia XR’s user interface, including its visual feedback system and game progression. Additionally, we clarify that Boccia XR uses a physics engine to simulate ball interactions within the projected game environment, ensuring realistic boccia dynamics.

**Revision:**

On page 11, lines 170–177, we added the following details about Boccia XR:

“Boccia XR does not currently incorporate sound or vibration feedback but provides immediate visual feedback by updating the projected image in real time. The system highlights the current throwing order and displays an indicator showing the position of the latest throw relative to previous throws, allowing participants to track game progress. Additionally, when a thrown ball collides with another ball in the projected image, a physics engine simulates the resulting movement based on predefined mechanics. This ensures that ball interactions between follow realistic Boccia dynamics while maintaining an intuitive and engaging experience.”

**Final Note:**

We sincerely appreciate the reviewer’s constructive feedback, which has strengthened our manuscript. We believe our revisions fully address your comments, but we remain available for further clarification if needed.

**Reviewer #3:**

**General Feedback:**

“Over all this is a very important work, congratulations.”

**Response:**

Thank you for your positive feedback. We appreciate your constructive comments, which have been valuable in refining our manuscript.

**Major Revisions**

**1. Introduction**

**Comment 1.1:**

“The introduction sets a clear rationale but could benefit from better alignment between the problem statement, research question, and hypotheses. Explicitly highlight the knowledge gap addressed by the study.”

**Response:**

Thank you for your insightful feedback. We have refined the Introduction to explicitly highlight the knowledge gap and strengthen the alignment between the problem statement, research question, and hypotheses. Specifically, we:

- Clarified the challenges older adults face in maintaining exercise adherence, emphasizing motivational and accessibility issues in conventional rehabilitation.
- Explicitly defined the research question, examining whether Boccia XR provides comparable or superior benefits to traditional Boccia and treadmill walking.
- Strengthened the connection between prior research and hypotheses to ensure a logical flow from the problem statement to study objectives.

These revisions improve the coherence of the Introduction and align the study rationale with its hypotheses.

**Revision:**

On page 5, line 71–72, we added:

“despite these advantages, Boccia is not widely implemented in rehabilitation settings due to its spatial requirements.”

On page 6, line 90–92, we added:

“However, the extent to which Boccia XR provides comparable or superior benefits to conventional Boccia and treadmill walking remains unclear.”

On page 7, line 99–101, we added:

“This study addresses this gap by examining the effects of Boccia XR on mood enhancement and lower limb muscle activity, comparing it with traditional Boccia and treadmill walking.”

**Comment 1.2:**

“While the hypotheses are mentioned, they should be more explicitly stated as testable predictions.”

**Response:**

We agree that explicitly stating the hypotheses improves clarity. We have reformulated them as testable predictions in the Introduction.

**Revision:**

On page 7, line 102–104, we corrected:

“We hypothesized that (1) both Boccia XR and traditional Boccia would improve mood more than treadmill walking, and (2) lower limb muscle activity would be partially higher in Boccia XR and traditional Boccia compared to treadmill walking.”

**2. Methods**

**Comment 2.1:**

“The sample size was calculated, but further clarification on the power analysis assumptions (e.g., expected effect size, variance estimates) is needed.”

**Response:**

Thank you for your comment. We have added details on the power analysis assumptions in the Methods section.

In accordance with statistical best practices for repeated-measures ANOVA, we report partial eta squared (ηp²) as a measure of effect size, as it accounts for within-subject variance. For non-parametric comparisons, we report Wilcoxon’s r, which is commonly used for rank-based tests. This ensures consistency in effect size reporting across different statistical methods.

**Revision:**

On page 8, lines 124–127, we corrected and added:

“A sample size calculation using G*Power software (Heinrich Heine University, Düsseldorf, Germany) determined that a minimum of 12 participants per condition was required. Further details on the power analysis are provided in the Statistical Analysis section.”

On page 20–21, lines 328–353, we added:

“To determine the appropriate sample size, an a priori power analysis was conducted using G*Power software. Previous studies on exergame interventions for older adults have reported moderate effect sizes (Cohen’s d = 0.54–0.65) in improving balance, physical activity, and psychological well-being [49-51]. However, some studies have raised concerns about usability and acceptance, suggesting that complex interfaces and unfamiliar technology may reduce motivation [52]. In contrast, Boccia XR is designed for intuitive use by older adults, eliminating the need for complex controllers or virtual environments. Its interactive and social nature is expected to enhance motivation and adherence in physical activity interventions. Given these advantages, we hypothesized that Boccia XR would have a greater impact than conventional exergames, potentially leading to a larger effect size. As no direct variance estimates were available from prior research, we adopted Cohen’s general guideline for behavioral intervention studies [53]. Based on this guideline, the sample size was calculated for a two-way repeated-measures ANOVA with an alpha level of 0.05, a power (1-β) of 0.80, and an effect size (f) of 0.40, which corresponds to a partial eta squared (ηp²) of approximately 0.14, classified as a large effect. The power analysis was based on expected differences in VA scores from POMS2, as mood enhancement was a primary outcome of interest. Given prior research on mood enhancement through physical activity, we anticipated a moderate to large effect size in VA scores, justifying the use of an effect size (f) of 0.40 for the analysis. The analysis indicated that a minimum of 12 participants per condition was required to achieve sufficient statistical power. Additionally, for comparisons conducted using the Friedman test, effect sizes were calculated using Wilcoxon’s r, following the formula:

r = Z / √N

The interpretation of Wilcoxon’s r followed Cohen’s criteria (0.1= small, 0.3= medium, 0.5= large), ensuring consistency in reporting effect sizes across different statistical methods.”

**Comment 2.2:**

“The order of tasks was randomized, but more details about the randomization method and potential carryover effects should be included.”

**Response:**

We have added details on the randomization method and addressed potential carryover effects in the revised manuscript.

**Revision:**

On page 9, lines 135–141, we corrected and added:

“Participants performed the XR, BO, and TR tasks at intervals of at least three days, with a maximum of two weeks to minimize potential carryover effects. While the minimum interval was set to three days to prevent residual effects from prior tasks, the actual washout period varied depending on participant availability and scheduling constraints. Block randomization was used to counterbalance task order. Participants were divided into three blocks of six, each beginning with a different task. Subsequent tasks were balanced within blocks to ensure even distribution.”

**Comment 2.3:**

“Differences in task duration (Boccia XR vs treadmill walking) could confound results. Address why this was not standardized.”

**Response:**

Thank you for your comment. Task duration was not standardized across conditions to preserve real-world gameplay dynamics in Boccia XR and conventional Boccia. Imposing a time constraint could have affected participants’ strategy and engagement. We have clarified this in the Methods section and discussed its potential impact as a study limitation.

**Revision:**

On page 19, lines 310–316, we added:

“Task duration was not standardized across conditions due to the inherent nature of each activity. While TR had a predefined duration for consistency, XR and BO followed a four-end game format against an examiner. To maintain ecological validity, these tasks were conducted under natural gameplay conditions without imposing an artificial time constraint. Standardizing the duration for Boccia tasks could have altered participants' strategic decision-making, engagement levels, and psychological responses, potentially affecting the effectiveness of the intervention.”

On page 31, lines 515–520:

“compared with treadmill walking, the duration and nature of the Boccia tasks differed. Boccia XR and traditional Boccia followed a natural gameplay format without a fixed duration, whereas treadmill walking was standardized to 360 seconds. While this design maintained ecological validity, differences in task duration may have influenced the results. Future studies should consider time-normalized analyses or standardized task durations to further clarify their impact.”

**3. Results**

**Comment 3.1:**

“Statistical results are presented, but effect sizes (e.g., Cohen's d or η²) are missing. Including these will improve result interpretation.”

**Response:**

We agree and have added effect sizes for all key statistical results, including partial eta squared for POMS and Wilcoxon’s r for EMG.

**Comment 3.2:**

“Highlight whether the observed changes in mood are clinically meaningful, not just statistically significant.”

**Response:**

We have addressed this by discussing the clinical significance of mood changes in the results section.

**Revision:**

On page 22–23, lines 368–373:

“The observed improvements in mood were statistically significant and also suggest clinical relevance, based on the classification thresholds provided in the POMS2 [41]. Post-intervention T-scores for VA and TMD fell within ranges associated with positive mood states, indicating potential psychological benefits. This effect was particularly evident in the XR and BO conditions, where VA scores showed greater increases and TMD scores demonstrated more substantial reductions.”

**4. Discussion**

**Comment 4.1:**

“The discussion restates results effectively but lacks depth in linking findings to broader literature.”

**Response:**

Thank you for your feedback. We have strengthened the Discussion by incorporating additional references and theoretical frameworks:

- We expanded the discussion on exercise motivation, integrating self-determination theory (SDT) to highlight how Boccia XR supports long-term adherence.
- We clarified the significance of muscle activity findings, emphasizing the role of quadriceps activation in fall prevention and mobility improvement.

**Revision:**

On page 25, line 413–419:

“Enjoyment is a crucial determinant of adherence to physical activity programs, particularly among older populations who often struggle to maintain motivation for regular exercise [45]. According to self-determination theory (SDT), intrinsic motivation plays a key role in sustaining physical activity, with autonomy, competence, and social relatedness being primary factors [20]. Boccia XR, by incorporating game-based elements and social interactions, aligns well with these motivational factors, potentially enhancing long-term adherence to exercise in older adults [22].”

On page 26–27, line 435–440:

“This RF activation is particularly important, as quadriceps strength plays a key role in fall prevention and mobility maintenance [39]. Quadriceps exercises have been shown to improve balance and reduce fall risk in aging populations [52]. Although MG activation was lower in Boccia XR compared to treadmill walking, similar trends have been reported in other task-oriented interventions that emphasize postural control over dynamic propulsion [29].”

**5. Figures and Tables**

**Comment 5**

Figure Clarity: Figures (e.g., EMG data) could benefit from clearer annotations or labels to make results more interpretable.

Table Readability: Ensure consistency in formatting across tables and clarify any ambiguous statistical notations.

**Response:**

We appreciate the reviewer’s suggestions regarding the clarity of figures and tables. To enhance transparency and consistency, we have revised the footnote in Table 2 to explicitly state that effect sizes (ηp², partial eta squared) for ANOVA results are reported in the text. Additionally, we have reformatted the footnote for improved readability while ensuring that all statistical information, including the application of the Bonferroni correction, is clearly presented.

Regarding the figures, we have improved the clarity and interpretability of Figure 3, we have made the following revisions:

- Added a title to the figure to provide a clear overview of the data.
- Displayed p-values only for statistically significant results to enhance readability and ensure clarity.
- Removed "trend-level significance (p < 0.1)" in response to another reviewer’s comment, ensuring consistency in statistical reporting.
- Included explanations of condition abbreviations in the footnote to facilitate understanding of the figure.

These modifications were made to enhance the figure’s readability and align with the reviewers' feedback.

**6. Ethical Considerations**

**Comment 6**

“Conflict of Interest: Explicitly address potential biases due to the involvement of an Institutional Review Board (IRB) chair as a co-author, even with a compliance certificate.”

**Response:**

Thank you for pointing out the importance of explicitly addressing ethical considerations. We have added a Competing Interests section after the Acknowledgements to clarify the role of the IRB chair as a co-author and to include a statement ensuring compliance with ethical standards.

**Revision:**

On page 32, lines 530–534, we have added a Competing Interests section and included the following statement.:

“The authors declare no competing interests. The Institutional Review Board chair involved in this study did not participate in the review process for this specific research protocol and was not involved in data collection or analysis to avoid any potential conflict of interest.”

**7. Overall Presentation**

**Comment 7.1:**

“Language and Grammar: The manuscript is generally well-written but could benefit from minor editing to improve readability and reduce redundancy.”

**Response:**

We appreciate your kind words and constructive feedback. We have made minor edits throughout the manuscript to improve readability and eliminate unnecessary redundancies. These revisions enhance the clarity of our discussion without altering the scientific content.

**Final Note:**

We sincerely appreciate your detailed and constructive comments, which have greatly improved our manuscript. We believe our revisions fully address your concerns but remain available for further clarification if needed.

**Reviewer #4**

**General Feedback:**

“The manuscript presents an innovative approach to rehabilitation for older adults using a gamified Boccia XR system, comparing its effects on mood and muscle activity to traditional Boccia and treadmill walking. While the study addresses a relevant topic and demonstrates novelty, it suffers from some methodological flaws... extensive revisions are necessary to strengthen the study design, analysis, and interpretation before reconsideration.”

**Response:**

Thank you for your detailed feedback and for recognizing the novelty and potential impact of our study. We appreciate your constructive suggestions and have made extensive revisions to address the methodological and interpretative concerns. Below, we provide point-by-point responses to each comment.

**Abstract**

**Comment 1:**

“l.33 Higher compared to what?”

**Response:**

Thank you for your comment. We have clarified the comparison target in the abstract. Additionally, in response to the following comment regarding Comment 2 “l.33 What is sufficient activity?”, we have revised the sentence to provide a clearer definition. The revised text explicitly states the reference point for comparison and clarifies what constitutes sufficient activity.

**Revision:**

On page 2–3, line 30-33, we corrected and added:

“Muscle activity analysis showed that Boccia XR and traditional Boccia involved muscle loads similar to treadmill walking, with rectus femoris activity greater than treadmill walking and medial gastrocnemius activity sufficient to aid muscle strengthening in sedentary older adults during Boccia tasks.”

**Comment 2:**

“l.33 What is sufficient activity?”

**Response:**

Thank you for your comment. In addressing this point, we have also clarified the comparison target mentioned in the previous comment. Specifically, we have revised the text to explicitly state the reference point for comparison and to define what constitutes "sufficient activity" based on existing guidelines for older adults.

**Revision:**

On page 2–3, line 30-33, we corrected and added:

“Muscle activity analysis showed that Boccia XR and traditional Boccia involved muscle loads similar to treadmill walking, with rectus femoris activity greater than treadmill walking and medial gastrocnemius activity sufficient to aid muscle strengthening in sedentary older adults during Boccia tasks.”

**Comment 3:**

“l.37 I believe you mean that it takes less space than a real boccia court, because it takes up more space than a treadmill.”

**Response:**

Thank you for this clarification. We have revised the text to specify this distinction.

**Revision:**

On page 3, line 35-37, we corrected:

“Boccia XR is an enjoyable, effective physical activity for older adults, requiring less space than a Boccia court but more than a treadmill. It provides physical benefits comparable to treadmill walking and may enhance exercise adherence and overall function in space-limited settings.”

**Introduction**

**Comment 4:**

“The introduction explains the problem well. It perhaps lacks some explanation of how they intend on comparing results.”

**Response:**

Thank you for your comment. We have clarified how the study compares results by explicitly stating that we examine the effects of Boccia XR on mood and muscle activity relative to traditional Boccia and treadmill walking. Mood changes were analyzed pre- and post-exercise, and muscle activity was measured during task execution. Given that these methodological details are already included in the Introduction, we believe they sufficiently address this concern.

**Revision:**

On page 7, line 99–101:

“This study addresses this gap by examining the effects of Boccia XR on mood enhancement and lower limb muscle activity, comparing it with traditional Boccia and treadmill walking.”

On page 7, line 104–107, we added:

“To test these hypotheses, we analyzed mood changes before and after exercise and measured muscle activity during task execution to determine whether Boccia XR is an effective as traditional Boccia.”

**Comment 5:**

“For example, why is treadmill walking monotonous? What are other sports used for rehabilitation and how do these compare to boccia?”

**Response:**

Thank you for your comment. We have clarified in the Introduction that treadmill walking is perceived as monotonous due to its repetitive nature, lack of environmental variation, and minimal social interaction.

While various sports, such as Tai Chi, aquatic therapy, and table tennis, have been utilized in rehabilitation settings, these activities often require specialized environments, equipment, or prior training. Boccia, on the other hand, is accessible, requires minimal equipment, and incorporates strategic elements that enhance engagement and cognitive stimulation. Given these characteristics, Boccia has potential as a rehabilitation activity for older adults. However, our study is not designed to compare Boccia with other rehabilitation sports, but rather to evaluate its effectiveness, particularly in its XR adaptation. We have clarified this in the Introduction.

**Revision:**

On page 4–5, line 56–58, we corrected and added:

“However, despite its benefits, treadmill walking is often perceived as monotonous due to limited environmental changes and minimal social interaction [16, 18].”

On page 5, line 66–71, we added:

“While various sports, such as Tai Chi, aquatic therapy, and table tennis, have been used in rehabilitation, they often require specific environments, specialized equipment, or prior training. In contrast, Boccia is accessible to a wide range of participants, requires minimal equipment, and incorporates strategic elements that enhance engagement and cognitive stimulation. These characteristics make Boccia a promising rehabilitation activity for older adults.”

**Comment 6:**

“l.70 You should explain what you mean by exercise load in the introduction.”

**Response:**

Thank you for your suggestion. We have explicitly defined 'exercise load' in the Introduction as lower limb muscle activity, measured using surface electromyography (EMG). This ensures consistency in terminology and highlights its relevance to Boccia XR’s effectiveness as a rehabilitation exercise.

**Revision:**

On page 7, line 93–99:

“In addition to enhancing motivation, an effective exercise program for older adults should provide sufficient lower limb muscle activation to maintain or improve physical function. In this study, exercise load specifically refers to the level of lower limb muscle activity during each task, assessed using surface electromyography (EMG). Given that lower limb strength is critical for mobility and fall prevention in older adults, evaluating muscle activation patterns in Boccia XR is essential to determine its potential as a rehabilitation exercise.”

**Comment 7**

“l.72 What is the goal of analysing muscle activity pre and post activity but not during?”

**Response:**

We realized the mistake in my writing thanks to your feedback. We appreciate your suggestion. Since muscle activity was measured during task execution, I have revised the relevant section accordingly.

**Revision**

On page 7, line 104–107, we added:

“To test these hypotheses, we analyzed mood changes before and after exercise and measured muscle activity during task execution to determine whether Boccia XR is an effective as traditional Boccia.”

**Methods**

**Comment 8:**

“l.80 What was the activity level of your subjects?”

**Response:**

Thank you for your comment. We have clarified the activity level of participants in the Participants section. They were recruited through the local Silver Human Resources Center, indicating engagement in daily activities such as part-time work or community service, though not necessarily in structured or high-intensity exercise. This information provides context for their general activity level and ensures that our findings are applicable to a broader population of healthy older adults.

**Revision:**

On page 8, line 116–119, we added:

“indicating their engagement in various daily activities, such as part-time work or community service, though not necessarily in structured or high-intensity exercise. Given their active lifestyle, they are representative of the general healthy older adult population.”

**Comment 9:**

“l.83 Power analyses are done on specific variables, what variable did you use to justify your sample size?”

**Response:**

Thank you for your insightful comment. We have clarified in the Statistical Analyses section that our power analysis was based on expected changes in Vigor-Activity (VA) scores from the Profile of Mood States 2nd Edition (POMS2). Given that mood enhancement was a primary outcome of our study, we used an anticipated effect size of 0.40 based on prior research on mood improvements through physical activity. This clarification ensures that our sample size justification is transparent and appropriately aligned with our study objectives.

**Revision:**

On page 21, line343–346, we added:

“The power analysis was based on expected differences in VA scores from POMS2, as mood enhancement was a primary outcome of interest. Given prior research on mood enhancement through physical activity, we anticipated a moderate to large effect size in VA scores, justifying the use of an effect size (f) of 0.40 for the analysis.”

**Comment 10:**

“l.89-l.93 These could be put into one paragraph for more fluid reading.”

**Response:**

Thank you for your suggestion. We have revised the relevant sections to combine the specified content into a single paragraph, improving readability and ensuring a more fluid narrative.

**Comment 11:**

“l.96 at least 3 and at most?”

**Response:**

Thank you for your question. We have clarified in the Interventions section that the interval between tasks was at least 3 days and typically up to two weeks. While the minimum interval was set at 3 days to prevent carryover effects, the washout period was generally longer, depending on participant availability and scheduling constraints. This ensures that residual effects from previous tasks were minimized, maintaining the validity of our comparisons.

**Revision:**

On page 9, line 135–139, we corrected and added:

“Participants performed the XR, BO, and TR tasks at intervals of at least three days, with a maximum of two weeks to minimize potential carryover effects. While the minimum interval was set to three days to prevent residual effects from prior tasks, the actual washout period varied depending on participant availability and scheduling constraints.”

**Comment 12:**

“There is confusion around who played against the subjects in each condition.”

“l.119-128 Did the opponent also play?”

**Response:**

We appreciate the reviewer’s suggestion to clarify the roles of opponents in each condition. We have revised the text to explicitly state that in both the Boccia XR and traditional Boccia conditions, participants played against an examiner who acted as an opponent while ensuring standardized gameplay interactions.

**Revision:**

On page 10, line 146–153:

“In both XR and BO, participants competed against an examiner who served as their opponent. The examiner maintained a consistent level of engagement across conditions to standardized gameplay interactions and minimize potential influence on participant performance. To ensure fairness and consistency, the examiner adjusted their level of play to match each participant’s ability, preventing extreme skill differences from affecting game outcomes. This approach ensured that the competitive aspect remained engaging while minimizing variability in participant performance due to opponent strength.”

**Comment 13:**

“l.124 Does it matter if they are close to the wall?”

**Response:**

Thank you for your question. We have clarified in the Interventions section that the 2.5 m distance from the wall was sufficient to prevent unintended interactions, such as upper limb contact with the wall or ball rebounds affecting gameplay. Additionally, no participants reported any issues related to wall proximity during the experiment. This ensures that the setup did not introduce unintended confounding factors.

**Revision:**

On page 12, line 182–185, we added:

“This distance was sufficient to prevent unintended interactions, such as upper limb contact with the wall or unexpected ball rebounds affecting gameplay. No participants reported issues related to proximity to the wall during the experiment.”

**Comment 14:**

“l.130 I recommend moving Fig 1 before l.119 as to not place multiple figures together.”

**Response:**

Thank you for your suggestion. We have revised the manuscript to move Fig 1 to an earlier position, ensuring that multiple figures are not placed together. This adjustment improves the visual organization of the manuscript and enhances readability.

**Comment 15:**

“l.147 Why this speed? This is also a follow up to the fitness level of your subjects.”

**Response:**

Thank you for raising this important point. In response to your suggestion, we have clarified our rationale for selecting the treadmill speed of 3.5 km/h. This speed was set slightly below 3.6 km/h (1.0 m/s), which is commonly regarded as the standard for independent ADL. Given that the participants in this study were independently ambulatory, this speed was chosen to ensure feasibility while also accommodating variability in fitness levels, including those whose walking speed may be slightly lower than this standard. This adjustment enhances the accessibility of the task while maintaining its relevance to the target population.

**Revision:**

On page 13, line 211–215, we have clarified the rationale for this speed as follows:

“This speed was set slightly below 3.6 km/h (1.0 m/s), which is commonly regarded as the standard for independent ADL. Since the participants in this study were independently ambulatory, this speed was chosen to ensure feasibility while also accommodating variability in fitness levels, including those whose walking speed may be slightly lower than this standard.”

**Comment 16:**

“l.161-178 You should explain this better. In the text you do not explain which parts pertain to the negative mood scales and which parts to the positive mood scales, implying that when the non-initiated reader reaches the equation, they do not understand why you are removing VA for a total score.”

**Response:**

Thank you for your feedback. We have revised the Mood State Measure section to clearly distinguish negative mood subscales (AH, CB, DD, FI, TA) from the positive mood subscale (VA). We also clarified that VA is subtracted in the Total Mood Disturbance (TMD) calculation because higher VA scores indicate greater vigor and energy, counterbalancing negative mood states. These revisions improve clarity for readers unfamiliar with POMS2.

**Revision:**

On page 15, line 237–247, we corrected and added:

“Among these subscales, AH, CB, DD, FI, and TA represent negative mood states, while VA represents a positive mood state. Friendliness (F) is not included in the Total Mood Disturbance (TMD) calculation. The TMD score is computed by summing the negative mood subscale scores (AH + CB + DD + FI + TA) and subtracting the VA score from this total. This method is used because higher VA scores indicate greater vigor and energy, which counterbalance negative mood states. Thus, a lower TMD score reflects an overall improvement in mood. The equation is as follows:

TMD score = total score of negative mood subscales (AH + CB + DD + FI + TA) – VA score.”

**Comment 17:**

“l.181-184 Why these muscles?”

“l.186 What do you consider the task for start and stop? Eight throws? From the beginning of the trial? Over one cycle? More detail should be added for the reader to appreciate the comparison between both sports which are not similar.”

“l.189 How were MVCs performed?”

“l.197 Does this mean that in walking you took the averaged activity post-processing over 1 minute and looked at its %MVC? Whereas in Boccia you looked at the average of eight throws. Were the times comparable?”

**Response:**

Thank you for your comments. We have substantially revised the EMG Data section for clarity, including:

- Muscle selection: Justifying the choice of muscles based on their roles in postural stability, mobility, and prior research.
- Task segmentation: Specifying the start and stop criteria for both Boccia and treadmill walking, including the analysis window.
- MVC measurement: Detailing standardization procedures, examiner-applied resistance, and non-elastic strap stabilization.
- %MVC normalization: Explaining how %MVC was calculated and addressing comparability between Boccia and treadmill walking.

While individual throw durations were not explicitly measured, %MVC normalization ensured meaningful comparisons across conditions despite movement structure differences.

**Revision:**

We have major revised the EMG data section.

On page 16, line 256–260:

“These muscles were selected for their essential roles in lower limb function, potential stability, and locomotion in older adults [46, 47]. They are commonly analyzed in studies on gait and postural control, making them suitable for comparing Boccia XR and treadmill walking [48].”

On page 16–19, line 263–303:

“Recording began before the examiner threw the jack ball and continued throughout the entire task session until all throw were completed.

Participants performed maximum voluntary contractions (MVC) of 3 seconds per muscle, with a rest interval of at least 1 minute between maximal efforts on each experimental day. MVC were performed in standardized postures against manual resistance provided by an examiner, with additional stabilization using non-elastic straps to minimize compensatory movements.

• Rectus femoris: Isometric knee extension in a seated position with 90° hip and knee flexion. The examiner applied resistance at the distal tibia, while both the thigh and pelvis were stabilized with straps to prevent hip movement.

• Semitendinosus: Isometric knee flexion in a prone position with the knee flexed to approximately 60°. The examiner applied resistance at the posterior distal tibia, while the pelvis was stabilized with a strap to prevent excessive hip movement.

• Tibialis anterior: Isometric dorsiflexion in a seated position with the knees at 90° flexion on the edge of a chair or bed. The examiner applied resistance at the dorsal aspect of the foot, while the lower leg was secured with a strap to minimize movement.

• Medial gastrocnemius: Isometric plantarflexion in a long-sitting position on a treatment table with fully extended knees. Participants leaned against a backrest for additional stability, and a non-elastic strap was placed around the pelvis to prevent compensatory trunk movement. The examiner applied resistance at the plantar aspect of the forefoot. A long-sitting position with a backrest was chosen to ensure participant stability during testing.

The recorded MVC values were used to normalize EMG signals (%MVC) for each task. For XR and BO, EMG analysis was performed post hoc, extracting data from the middle eight throws (throws 9–16) to minimize variability due to initial adaptation and potential fatigue effects. The analysis window for each throw began when the participant assumed the throwing stance at the throwing line and ended when they returned to a static standing position after releasing the ball.

%MVC was calculated for each of the middle eight throws (throws 9–16), and the average %MVC was then computed across these throws for each participant. While the exact duration of each throw was not recorded, %MVC normalization ensured meaningful comparisons across conditions despite differences in movement structure.

For treadmill walking, EMG signals were analyzed during the middle 3 minutes of the 6-minute walking period to avoid transient changes in muscle activity at the beginning and end of the trial. A root mean square (RMS) filter (100 ms window) was applied to the EMG signals to smooth the data.

For each task, movement time was normalized to 100% to allow for consistent comparisons across conditions.”

**Comment 18:**

“l.191 Why were only 8 throws analysed per condition?”

**Response:**

Thank you for your question. We selected the middle eight throws (throws 9–16 out of 24) to minimize adaptation and fatigue effects. Initial throws may involve adaptation, while later throws could be influenced by fatigue. Analyzing the middle phase provides a stable representation of muscle activity.

Using a fixed number of throws also ensures consistency across participants and conditions, allowing for reliable comparisons. We have clarified this rationale in the Methods section.

**Revision:**

On page 18, line 289–291:

“For XR and BO, EMG analysis was performed post hoc, extracting data from the middle eight throws (throws 9–16) to minimize variability due to initial adaptation and potential fatigue effects.”

**Results:**

**Comment 19:**

“l.226 significant tendency.”

**Response:**

Thank you for your comment. To maintain statistical rigor and adhere to common reporting standards, we have removed references to 'trend-level significance (p < 0.1)'. While some results approached significance (e.g., p = 0.06), we acknowledge that these findings require cautious interpretation.

We now describe these results as "not statistically significant but suggestive of a potential trend", and we discuss the need for further investigation in future studies. These changes ensure that we present the data objectively while acknowledging meaningful patterns that warrant further exploration.

**Revision:**

We corrected follows:

On page 22, line 359:

“but it did not reach statistical significance”

On page 23–24, line 384–385:

“was higher than in TR, but the difference was not statistically significant”

On page 26, line 422–426:

“Although the interaction effect for VA scores did not reach statistical significance (p = 0.07), post hoc analyses indicated significant improvements in VA scores after XR and BO tasks. This suggests that Boccia XR and traditional Boccia may have a positive impact on mood, but further studies with larger sample sizes are needed to confirm this effect.”

On page 29, line 482: “indicating”

On page 29, line 483: “are unlikely to induce”

**Comment 20:**

“ l.228 what are the values for the Bonferroni correction?”

**Response:**

Thank you for your question. We have clarified the application of the Bonferroni correction in both the Statistical Analysis section and the footnote of Table 2. Specifically, for multiple comparisons in the POMS2 data analysis, the Bonferroni correction was applied, setting the corrected significance threshold as α = 0.05 / 3 = 0.0167. Pairwise comparisons were considered statistically significant only if the Bonferroni-adjusted p-value was less than 0.0167. This clarification has been explicitly included in the revised manuscript.

**Revision:**

On page 20, line 323–325, we added:

“Bonferroni correction was applied for multiple comparisons, adjusting the significance level to α= 0.05 / 3= 0.0167 to control for Type I error inflation.”

On page 23, line 379–380, we added:

“Bonferroni correction was applied for multiple comparisons, adjusting the significance level to α= 0.05 / 3= 0.0167.”

**Comment 21:**

“l.242 no statistical difference between XR and TR it should not be specified in a figure.”

**Response:**

We have revised the figure to focus on statistically significant results. Additionally, we have presented the muscle activity levels and comparisons between conditions that showed no significant differences in the Results section.

**Revision:**

Figure 3 has been updated to exclude non-significant comparisons between XR and TR.

On page 24, line 390–395, we added:

“ST activity did not differ significantly among conditions (XR: 14.0, BO: 16.2, TR: 14.9 %MVC; XR vs. TR: Wilcoxon’s r = 0.078, p > 0.05; BO vs. TR: Wilcoxon’s r = 0.078, p > 0.05; XR vs. BO: Wilcoxon’s r = 0.16, p > 0.05). Similarly, TA activity did not differ significantly among conditions (XR: 20.2, BO: 21.0, TR: 22.3 %MVC; Wilcoxon’s r = 0 for all comparisons, p > 0.05).”

**Discussion**

**Comment 22:**

“ l.261 Please read my comments about multiple participants in the methods and add a part about this here.”

**Response:**

Thank you for your insightful comment. We have clarified this point in the Discussion section. While our study design did not involve multiple participants playing together, participants engaged in one-on-one gameplay against an examiner. The examiner’s role was standardized to ensure consistency across participants; however, natural interactions, such as verbal exchanges and encouragement, occurred during gameplay. These interactions may have contributed to increased motivation and enjoyment, aligning with previous research suggesting that social engagement during structured physical activities can enhance emotional well-being. We have revised the manuscript accordingly to reflect this perspective.

**Revision:**

On page 27, line 446–451, we added:

“Although our study design did not involve multiple participants playing together, participants engaged in one-on-one gameplay against an examiner. The examiner’s role was standardized to maintain consistency across participants; however, even within this structured format, natural interactions occurred during gameplay. Verbal exchanges, encouragement, and the dynamic nature of competition may have contributed to increased motivation and enjoyment.”

**Comment 23:**

“l.266 POMS2 measures for positive mood changes and describes VA as a vigorous or energetic measure but not enjoyment. Is this speculation?”

**Response:**

Thank you for your valuable feedback. We have revised the manuscript to clarify the relationship between Vigor-Activity (VA) and enjoyment in physical activity. While VA primarily measures energy and enthusiasm, previous research suggests that positive emotional states are closely linked to enjoyment in exercise. We have incorporated the broaden-and-build theory to support this connection, emphasizing that positive emotions can enhance engagement and long-term adherence to physical activity. Additionally, we have modified the Introduction section to explicitly state that VA is used as an indicator of positive emotional engagement, which may contribute to overall enjoyment. These revisions ensure that our interpretation aligns with existing literature while maintaining the focus on enjoyment as a key outcome of this study.

**Revision:**

On page 4, line 49–53:

“Enjoyment in physical activity is influenced by multiple factors, but prior research suggests that positive emotional states, such as increased vigor, are closely associated with perceived enjoyment [10]. Based on this, we assess enjoyment by evaluating positive mood changes during exercise.”

On page 26, line 426–433:

“While VA primarily reflects energy and enthusiasm, previous research suggests that positive emotional states, such as increased vigor, are closely associated with the perception of enjoyment in physical activity [10]. Fredrickson’s broaden-and-build theory posits that positive emotions enhance engagement and long-term adherence to activities by expanding cognitive and behavioral repertoires [45]. Based on this theoretical framework, our findings suggest that Boccia XR and Boccia, by increasing vigor and reducing negative mood, may contribute to a more enjoyable exercise experience compared to treadmill walking.”

**Comment 24:**

“l.274 Most muscles shown above (fig 3) did not reach 25-30% of MVC (apart from TA). Does this suggest that none of your tasks are sufficient for muscle hypertrophy?”

**Response:**

Thank you for your comment. While Boccia XR and Boccia (except for TA) did not reach hypertrophy thresholds, they may still benefit neuromuscular function, endurance, and postural stability, which are essential for older adults. We have revised the Discussion section to clarify this point.

**Revision:**

On page 29–30, line 481–494:

“In our study, the muscle activation levels observed in XR and BO (except for TA) were below this threshold required for hypertrophy, indicating that these activities are unlikely to induce significant muscle growth. However, previous studies have shown that even low-intensity resistance exercises can improve neuromuscular function in older adults, particularly those with reduced physical activity [65, 66]. While our findings suggest that Boccia XR and traditional Boccia may provide benefits for lower limb function, further studies are needed to determine their long-term impact on muscle conditioning. Given that Boccia XR and traditional Boccia involve repeated lower limb engagement, they may still contribute to muscle endurance, neuromuscular coordination, and postural stability, which are essential for mobility and fall prevention in aging populations. Thus, although Boccia XR and traditional Boccia are not designed as hypertrophy-oriented resistance training, they may serve as effective rehabilitation exercises for maintaining lower limb function and enhancing exercise adherence in older adults.”

**Comment 25:**

“l.282 You present task duration in both the methods and the results section, was the point only to say that this takes around 15 minutes?”

**Response:**

Thank you for your question. We have clarified the purpose of reporting task duration in both the Methods and Results sections. In the Methods section, we specify the measurement procedure to ensure consistency across participants and account for potential differences in physical exertion. In the Results section, we report the actual task completion times and highlight their relevance, particularly the feasibility of Boccia XR as a short-duration exercise for older adults. This clarification strengthens the rationale for including task duration in both sections.

**Revision:**

On page 19, line 306–308, we added:

“to ensure consistency across participants and to account for potential differences in physical exertion among conditions.”

On page 25, line 405–407, we added:

“Boccia XR required approximately 14 minute to complete, making it a relatively short-duration activity compared to traditional rehabilitation exercise.”

**Data Availability**

**Comment 26:**

“l.300 the authors say this is available per reasonable request. This should either be specified as to why they do not want to share per the regulation or publish the data.”

**Response:**

We have revised the data availability statement to comply with PLOS ONE policies.

**Revision:**

On page 32, line 537: “All relevant data are available within the Supporting Information files.”

**Final Note:**

We sincerely appreciate your thorough review and constructive feedback, which have greatly improved our manuscript. We believe our revisions fully address your concerns but remain available for further clarification if needed.

**Reviewer #5**

**General Feedback:**

“The study used a sample of 18 healthy elderly participants. For the comparison between two different types of Boccia activities and treadmill walking, this sample size seems small... extensive revisions are necessary to strengthen the study design, analysis, and interpretation before reconsideration.”

**Response:**

Thank you for your feedback and for highlighting areas for improvement. We have addressed each comment and revised the manuscript accordingly. Below are our detailed responses.

**Comments and Responses**

**Comment 1:**

“The study used a sample of 18 healthy elderly participants. For the comparison between two different types of Boccia activities and treadmill walking, this sample size seems small. A small sample may lead to insufficient statistical power. Moreover, the study may be affected by the differences and diversity of the participants, so the universality and reliability of the conclusions are questionable. Besides, short-term experiments cannot verify the impact of Boccia XR on long-term exercise adherence.”

**Response:**

We acknowledge the limitations of the small sample size and short study duration. These have been explicitly discussed in the revised manuscript, along with recommendations for future studies with larger and more diverse samples.

**Revision:**

On page 30–31, lines 504–512, we included:

“The small sample size (n=18) limits the statistical power and generalizability of the results. Additionally, the study included only healthy older adults, which may limit the external validity of the findings, particularly for populations with different health conditions or functional limitations. Furthermore, none of the participants had prior experience playing Boccia, which may have minimized variability in baseline skill levels but could also have influenced familiarity with the game. Future studies with larger and more diverse populations, including individuals with varying health conditions, are needed to validate these findings and explore the long-term effects of Boccia XR on exercise adherence.”

**Comment 2:**

“The study only selected healthy elderly individuals, which may limit the application of the results to elderly people with different health conditions or functional limitations. It is recommended to consider including elderly people with different health conditions to improve the external validity of the results.”

**Response:**

Thank you for your suggestion. We agree that including only healthy elderly individuals may limit external validity. Since this relates to sample size and study duration, we have addressed it in the same section discussing these factors for clarity and conciseness.

We note that a similar concern was raised regarding sample size and the duration of the intervention. As a result, we have addressed both comments with the same revision, integrating these limitations into a unified discussion.

**Revision:**

On page 30-31, lines 507–515, we included:

“The small sample size (n=18) limits the statistical power and generalizability of the results. Additionally, the study included only healthy elderly individuals, which may limit the external validity of the findings, particularly for populations with different health conditions or functional limitations. Furthermore, none of the participants had prior experience playing Boccia, which may have minimized variability in baseline skill levels but could also have influenced familiarity with the game. Future studies with larger and more diverse populations, including individuals with varying health conditions, are needed to validate these findings and explore the long-term effects of Boccia XR on exercise adherence.”

**Comment 3:**

“Although the study mentioned performing different tasks in a random order, it did not elaborate on the randomization method and how to control potential confounding variables, such as the participants' individual differences in responses to different tasks. The lack of detailed randomization and control strategies may lead to bias in the results.”

**Response:**

Thank you for your observation. We have added details on the randomization method and strategies to minimize confounding variables.

**Revision:**

On page 9, lines 139–141, we added:

“Block randomization was used to counterbalance task order. Participants were divided into three blocks of six, each beginning with a different task. Subsequent tasks were balanced within blocks to ensure even distribution.”

**Comment 4:**

“The experimental environments of Boccia XR and traditional Boccia are described in relatively detailed ways, but the description of the environment for treadmill walking is insufficient. For example, it is not clear whether the tests were carried out on different days or time periods, which may affect the participants' performance. It is recommended to add relevant explanations.”

**Response:**

We agree that the treadmill walking environment required clarification. We have specified that all sessions were conducted in a controlled laboratory environment under consistent conditions.

**Revision:**

On page 13, lines 209–210:

“TR was performed in a controlled laboratory environment under consistent experimental conditions.”

**Comment 5:**

“The study reported that Boccia XR and traditional Boccia are significantly better than treadmill walking in enhancing positive emotions. This may be due to the fun brought by social interaction and the nature of games. However, considering individual differences in emotional states and the order effect between tasks, this result needs to be interpreted more cautiously.”

**Response:**

Thank you for your comment. We have revised the discussion to acknowledge that individual differences in emotional states and order effects may have influenced the results. While task order was randomized, residual effects cannot be entirely ruled out. We suggest future studies use fully counterbalanced designs to better control for these variables.

**Revision:**

On page 28, line 456–460, we added:

“However, individual differences in baseline emotional states and potential order effects may have influenced these findings. Although task order was randomized to minimize biases, residual effects cannot be entirely ruled out. Future studies should incorporate fully counterbalanced designs to further control for these variables and strengthen the validity of the results.”

**Comment 6:**

“The study results show that some muscle activity levels of Boccia XR and traditional Boccia are similar to those of treadmill walking. However, there is not enough information to explain why some muscle activities (such as MG) are higher during treadmill walking. A more detailed analysis of the possible reasons for these data differences is needed in the discussion.”

**Response:**

Thank you for your comment. We have expanded the discussion to explain why MG activity was higher during treadmill walking.

**Revision:**

On page 28–29, lines 473–475:

“The higher MG activity observed during TR likely reflects the continuous engagement of MG for propulsion and balance. In contrast, Boccia tasks involve intermittent muscle activation patterns, which may explain these differences.”

**Final Note:**

We sincerely appreciate your constructive feedback, which has significantly improved our manuscript. We believe our revisions fully address your concerns but remain available for further clarification if needed.

**Final Statement**

We sincerely appreciate the time and effort the reviewers and editors have dedicated to evaluating our manuscript. Their insightful comments have greatly contributed to improving the clarity, rigor, and overall quality of our study. We believe that the revised manuscript now more effectively communicates our findings and their significance.

We hope that these revisions fully address all concerns, and we look forward to the opportunity to contribute to the journal. Please do not hesitate to reach out if further clarifications are needed. Thank you for your consideration.
